# Extreme metabolic alkalosis in intensive care

**DOI:** 10.4103/0972-5229.60175

**Published:** 2009

**Authors:** Swagata Tripathy

**Affiliations:** **From:** Department of Anesthesia and Intensive Care, Kalinga Institute of Medical Sciences Medical College, Bhubaneswar, India

**Keywords:** Extreme metabolic alkalosis, respiratory compensation, treatment

## Abstract

Metabolic alkalosis is a commonly seen imbalance in the intensive care unit (ICU). Extreme metabolic alkalemia, however, is less common. A pH greater than 7.65 may carry a high risk of mortality (up to 80%). We discuss the entity of life threatening metabolic alkalemia by means of two illustrative cases - both with a pH greater than 7.65 on presentation. The cause, modalities of managing and complications of this condition is discussed from the point of view of both the traditional method of Henderson and Hasselbalch and the mathematical model based on physiochemical model described by Stewart. Special mention to the pitfalls in managing patients of metabolic alkalosis with concomitant renal compromise is made.

## Introduction

Extreme metabolic alkalemia has been associated with a high risk of mortality of up to 45% with a pH of 7.55 and 80% when pH is greater than 7.65. Appropriate intervention and correction is warranted when arterial blood pH exceeds 7.55.[1]

We discuss two cases who presented to our intensive care unit with extreme metabolic alkalemia (pH 7.65). One was managed with simple replacement measures. The other defied even better known interventions for management of alkalemia. We discuss the manifestations, causes and management of ‘extreme’ life threatening alkalemia.

## Case Reports

A 60-year-old man presented to the emergency department with the complaints of weakness, difficulty in breathing, decreased appetite and absolute constipation for ten days. He was restless and disoriented. Hemodynamic parameters were normal. Investigations and systemic examination revealed severe metabolic alkalosis (pH 7.66) with dyselectrolytemia and volume depletion [Tables 1 and 2]. His respiratory and neurologic systems were unremarkable. The abdomen was distended and multiple fluid and air levels seen on an abdominal radiogram, confirmed sub acute intestinal obstruction.

**Table 1:** Parameter on admission of patient 1 and 2w

Body weight (kg)	68	54
Height (cm)	164	168
Temperature (°F)	98.8	102.6
Blood pressure (mmHg)	100/70	100/60
Heart rate (beats/min)	90	100
Respiratory rate (breaths/min)	10	30
Haemoglobin (gm/dl)	14.4	8.6
Venous plasma		
Chloride (mEq/l)	70	40
Potassium (mEq/l)	2.6	1.0
Sodium (mEq/l)	106	100
Calcium [mg/dl]	8.5	6.0
Magnesium [mg/dl]	1.8	-
Phosphate [mg/dl]	2.8	3.8
Urine		
Chloride (mEq/dl)	12	8
pH	6.6	6.0
Urea [mg/dl]	29	64
Creatinine [mg/dl (μmol/l)]	0.7	3.4
Albumin (g/dl)	2.9	2.4
Electrocardiography	Normal	LVH
Chest radiography	Normal	cardiomegaly
Computer Tomogram (head)	-	Normal

**Table 2:** Arterial Blood Gas values

Patient 1	P1_ADMN_	P1_DAY 3_	Patient 2	P2_ADMN_	P2_DAY 4_
PaO_2_ (FiO_2_)	85 (0.5)	70 (0.3)	PaO_2_ (FiO_2_)	80 (0.3)	110
PaCO_2_	38	42	PaCO_2_	40	52
HCO_3_-	41	28	HCO_3_-	45	62
pH	7.66	7.46	pH	7.69	7.7
BE	19.3	5	BE	24.7	39.1

Despite denials of use of any diuretics or laxatives, when his regular medicines were checked, loop diuretics and combinations with potassium sparing diuretics were recovered-confirming long term diuretic abuse. All diuretics were removed from patient's bedside and patient was hydrated to achieve a central venous pressure (CVP) of at least 10 cm water (opening CVP 1 cm water) and a urine output of more than 1 ml/kg/hour. Obstipation was relieved by manual evacuation of hard dried up faeces - the cause of sub acute intestinal obstruction.

Supplementation of potassium, chloride, sodium and magnesium led to improvement of serum electrolytes and resolution of alkalemia over 36 hours [Table 2]. The patient's respiratory distress, weakness and lassitude resolved. Patient was discharged after four days without any complains.

### Patient 2

A 50-year-old man with past history of hypertension and chronic renal failure was admitted to our hospital complaining of high grade fever, vomiting and diarrhoea for the past week. On examination he was lethargic, with slow eye photomotor reflexes, confused on arousal, with poorly coordinated movements and speech. He appeared malnourished, dehydrated, and oliguric. The relevant laboratory investigations and clinical picture reflected severe chloride-potassium-fluid depletion with metabolic alkalosis (pH 7.69), compensatory hypoventilation, hypercapnia, hypoxemia and acute-on-chronic renal failure [Tables 1, 3]. Computer tomography of the brain was normal.

Under careful monitoring, replacement of normal free fluid, potassium and sodium chloride was started. Milk based enteral nutrition (up to 2 litres per day) along with supplementation of magnesium, phosphate, vitamins and trace elements were started.

Urinary tract infection (*E. coli* growth in urine) was treated with antibiotics according to sensivity reports. Inspite of improvement in presenting complains of fever, vomiting and diarrhoea, his renal function deteriorated progressively over the next two days (serum urea 90 mg/dl, creatinine 5.2 meq/dl). Extreme alkalemia persisted. Oral acetazolamide 250 mg per day was started and the frequency of the proton pump blocker was increased to twice a day. Haemodialysis with dialysate containing 25-28 meq/dl of bicarbonate was tried twice. Lower bicarbonate containing dialysate was unavailable. Intravenous calcium gluconate (10 mg) followed by 1500mg calcium carbonate per day was administered for hypocalcemia (corrected serum calcium levels 7 gm/dl). On the fourth day he developed further deterioration in level of consciousness and developed myoclonic jerks, tetany and seizures. CT scan revealed an acute subdural hemorrhage in the right parietal region. Procurement of medical grade hydrochloric acid was attempted. However, patient was shifted to a government hospital due to financial constraints where he expired after two days.

## Discussion

Metabolic alkalosis has been classified traditionally as chloride responsive and chloride non responsive varieties. Most severe metabolic alkalosis is of the chloride-responsive form (as in both our patients), the common causes being loss of gastric acid and the administration of loop or thiazide diuretics.[2] Extreme alkalemia (blood pH greater than 7.65) carries a high risk of complications [Table 3].

**Table 3:** Major adverse consequences of severe alkalemia

Cardiovascular
Arteriolar constriction
Reduced coronary blood flow
Reduced anginal threshold
Predisposition to refractory arrhythmias
Respiratory
Hypoventilation with hypercapnia and hypoxemia
Metabolic
Stimulation of anaerobic glycolysis
Organic acid production
Hypokalemia
Decreased plasma ionized calcium concentration
Hypomagnesemia and hypophosphatemia
Cerebral
Reduction in cerebral blood flow
Tetany, seizures, lethargy, delirium, and stupor

Two methods are used for diagnosing and interpreting acid base data.[3] The Henderson and Hasselbalchs equation, the traditional method, is based on serum bicarbonate level and the anion gap. The other is based on the physicochemical model of Stewart. The former if applied in our cases would result in the diagnosis of a pure metabolic alkalosis in the first case. A coexistent metabolic acidosis and alkalosis in the second case would be apparent only after correction for hypoalbuminemia.

The Stewarts model proposes three variables that independently determine the concentration of hydrogen ions (H^+^) and, consequently, the pH. These variables are the strong ion difference (SID): difference between fully dissociated anions and cations, practically [Na^+^-Cl^−^], the total weak acid concentration (especially albumin and phosphate) (A_tot_) and PaCO2.[4] In Stewarts analysis of alkalosis, contraction alkalosis can be seen in the patient who has been fluid restricted or treated with diuretics. Treatment of contraction alkalosis simply requires free water administration in the form of hypotonic solutions.

Chloride shifts occur in relation to gastrointestinal abnormality. If the hyperchloric gastric contents are lost through vomiting or through gastric tube suction then a hypochloremia can result. Hypochloremia leads to an increase in SID. The positive charge increase associated with the SID must be balanced by a reduction of H^+^ ionization to keep the charges equal. Reduced ionisation of H^+^ leads to a rise in pH. Administration of normal saline constitutes effective treatment. This treatment can be illustrated in the same fashion as free water changes (case 2, explained later).

Hypoalbuminemia is a common finding in critically ill patients. It may confound interpretation of acid base abnormalities by the routine HCO^3−^ methods because it is a major nonbicarbonate buffer in the blood plasma along with Pi.[3] Stewarts method gives an emphasis on the serum albumin levels, emphasising the contribution of hypoalbuminemia to metabolic alkalosis as seen in both our cases. The anion gap (AG) is also effective in elucidating hidden disorders when corrected for serum albumin levels.[5]

It is now possible to partition the base deficit at the bedside with enough accuracy to permit clinical use by using the abbreviated Fencl Stewart equations for strong ions and albumin.[6,7] This provides valuable information on the aetiology of acid-base disturbance. The formulae involved are derived from the more complicated Stewarts equations, and we will use them to analyze our examples. The details are outside the scope of this article: these are:

BDtot - BDalb - BD_Cl_ = BD_UMA_[1]

BD_alb_ = (42 - albumin (g/L)) × 0.25[2]

BD_Cl_ = [Na^+^] - [Cl^−^] - 32[3]

SID = Na - Cl (SID <38 is low SID metabolic acidosis)[4]

Corrected Chloride = Cl × [Na normal/ Na observed]

If the primary problem is metabolic alkalosis

Expected ↑ CO_2_ = 0.6 × Base Excess[5]

(BD_alb_, base deficit due to albumin; BD_Cl_, base deficit due to chloride; BD_tot_, total base deficit; BD_UMA_, base deficit due to unmeasured anions; SID, Strong Ion Difference.)

A BD_UMA_ less than −5 meq/dl is associated with greater mortality than a high anion gap or serum lactate level. Elevated unmeasured anions identified by the Fencl-Stewart method were more strongly associated with mortality than with BE, AG, or lactate.[8]

### Patient 1

In this patient, the SID = 106−70= 36. Reduction of SID by 2 mEq/dl and low SID metabolic acidosis results mainly from plasma water excess possibly due to drinking of free water or 5% dextrose administration to correct dehydration. As the corrected Cl is [70× 140/106 = 92.4] within normal limits, there is no hypochloremia even though it may appear so. This low SID metabolic acidosis is hidden by the alkalinising hypoalbuminemia [a positive BD_UMA._ As BD alb = (42 − 29) × 0.25 = 3.25, BD cl = 106 − 70 − 32 = 4 and BE tot = 19] and inadequate respiratory compensation for metabolic alkalosis (expected rise in CO_2_ is 0.6 × 19 = 11.4 i.e., CO_2_ should be 51.4, but actually is 38) possibly due to anxiety or pain.

Severe dehydration in the first patient led to subacute intestinal obstruction. The alkalosis was reversible with relative ease by rehydration and potassium supplementation.

Loss of sodium, chloride and fluids caused by diuretics promote metabolic alkalosis by several possible mechanisms:[9] 1) Diuretic-induced increases in sodium delivery to the distal nephron accelerate potassium and proton secretion; 2) ECF volume contraction stimulates renin and aldosterone secretion, which blunts sodium loss but accelerates the secretion of potassium and protons. Halperin *et al.* have given a succinct example of how a contraction of ECF volume form 10 L to 4 L would cause a rise in serum bicarbonate from 25 mmol/L to 62 mmol/L even with no external gain/loss of bicarbonate; 3) potassium depletion will independently augment bicarbonate reabsorption in the proximal tubule; and 4) stimulate ammonia production, which, in turn, will increase urinary net acid excretion; 5) hypoalbuminemia contributes to the alkalosis. Despite the severity of the disorder it was not lethal, and recovery was attained by carefully tailored replacement therapy under strict monitoring, without the need for other emergency therapies.

### Patient 2

In this patient, there is a high SID metabolic alkalosis, with corrected hypochloremia, implying dehydration and contraction alkalosis (SID = 100 − 40 = 60; Cl corr = 40× 140/100 = 56). This is also enhanced by the hypoalbuminemic alkalosis.

However, this patient being one of renal failure would also be expected to have metabolic acidosis, which is apparent with the calculation of Fencl Stewarts BE_UMA_

BE_UMA_ in this case is −7.8 implying metabolic acidosis to unmeasured anions (BE alb 42-24/4=4.5; BE cl = 100 − 40 − 32 = 28; BE tot = 24.7)

This patient has an expected CO2 of (0.6 × 24.7 = 14.8) which is also not adequately compensated possibly due to the fever, thereby adding to the alkalemia.

Another point of interest is the highly negative BE_UMA_ in this case which also correlates eventually with this patient's poor prognosis.

When patients of renal failure have superimposed electrolyte imbalance and alkalemia as in our second case, management with replacement and haemodialysis (an alkalising process) can be a considerable challenge.[10] Alkalosis is unusual in patients with advanced renal failure due to coexisting cation accumulation. However, external alkali load may accumulate rapidly due to impaired alkali secretary capacity.

The postulated causes of persistence of alkalemia and our failure to treat it adequately are:

1. Inadequate respiratory compensation - Fever and later acute SDH probably prevented hypoventilation and compensation. Elective ventilation and permissive hypercarbia might have been a heroic attempt at achieving a more acceptable blood pH had we not underestimated the refractoriness of the alkalemia.
2. The milk based feeds and calcium supplementation given for hypocalcemia induced tetany probably caused a ‘milk alkali syndrome’.[11]
3. The intermittent hemodialysis may have worsened the condition.
4. Hypoalbuminemia potentiated the alkalosis.

Although conventional hemodialysis can correct severe alkalemia and volume overload,[12,13] special low bicarbonate baths may be more helpful in managing severe alkalemia.

The same goals can be achieved by continuous arteriovenous or venovenous hemofiltration with sodium chloride as the replacement solution.

Loss of gastric acid can be reduced by administering H_2_-receptor blockers or proton pump inhibitors, which substitute loss of sodium chloride for loss of hydrochloric acid.[14] Acetazolamide has been seen to be rapidly effective in normalizing base excess. It is more readily available than sterile hydrochloric acid or ammonium chloride.[15] Its renal site of action may render it ineffective patients with end stage renal disease. High doses may cause prolonged neurologic disturbance in these patients.[16]
